# Impact of Harvest Time on the Dry Matter Content, and Nutritional Parameters Related to Forage Quality of Maralfalfa (*Cenchrus purpureus* (Schumach.) Morrone, Poaceae) under Mediterranean Climate

**DOI:** 10.3390/plants12234045

**Published:** 2023-11-30

**Authors:** Joaquín Fayos-Febrer, Jorge Juan-Vicedo, Alba Rodríguez-Mengod, Javier Mazón, Juan Carlos Gardón

**Affiliations:** 1 Departamento de Biotecnología, Facultad de Veterinaria y Ciencias Experimentales, Universidad Católica de Valencia, Carrer Guillem de Castro, 94, 46001 València, Spain; jfayos@upv.es; 2 Instituto de Investigación en Medio Ambiente y Ciencia Marina IMEDMAR, Universidad Católica de Valencia, Carrer Guillem de Castro, 94, 46001 València, Spain; jorge.juan@ucv.es; 3 Departamento de Producción Animal y Salud Pública, Facultad de Veterinaria y Ciencias Experimentales, Universidad Católica de Valencia, Carrer Guillem de Castro, 94, 46001 València, Spain; alba.rodriguez@ucv.es; 4 Departamento de Medicina y Cirugía Animal, Facultad de Veterinaria y Ciencias Experimentales, Universidad Católica de Valencia, Carrer Guillem de Castro, 94, 46001 València, Spain; j.mazon@colvet.es

**Keywords:** maralfalfa, *Cenchrus purpureus*, amino acids, forage, fodder crops, nutritional, harvest time

## Abstract

Maralfalfa (*Cenchrus purpureus* (Schumach.) Morrone) is a productive fodder crop in tropical regions that has been evaluated for forage nutritional value in a Mediterranean climate. To assess the nutritional value, parameters including dry matter content (DM), ash, ether extract (EE), protein (CP), fiber contents (NDF and ADF), and the amino acids profile were determined at eight harvest times (HTs) in a non-fertilized and non-irrigated crop based in Silla (Valencia, Spain). The results showed significant differences in most of the parameters studied. While CP and ash significantly decreased over the eight HTs, NDF and ADF increased. In contrast, EE and the ratio of essential amino acids/total amino acids remained constant. Values of CP remained higher than 15% during the first two HTs (16 and 28 days). According to the analyses performed, the optimum HT can be stated at 28 days as it combines high levels of CP (including an optimal combination of essential amino acids) with low levels of fibers (NDF = 57.13%; ADF = 34.76%) and a considerable amount of dry matter (15.40%). Among the essential amino acids (EA) determined, lysine and histidine showed similar values (Lys ≈ 6%, His ≈ 1.70%) when comparing the composition of these EA to other forage species and cultivars studied, whereas methionine showed lower values. This work establishes the basis for the appropriate HT of maralfalfa according to the nutritional parameters measured. Further studies could be aimed to optimize the nutritional and phytogenic properties of maralfalfa to improve its value as a fodder crop, and to finally introduce it for sustainable livestock production in Mediterranean countries.

## 1. Introduction

*Cenchrus* L. (previously known as *Pennisetum* Rich.) is a genus belonging to the Poaceae family, and some of its species represent an important source of perennial herbaceous fodder crops in tropical regions [1,2]. Specifically, *C. purpureus* (Schumach.) Morrone, popularly known as maralfalfa, has been the subject of interspecific hybridization-based plant breeding programs to develop highly productive forages that can be adapted to several types of environments in tropical and dry regions all over the world [1,2,3,4,5].

On one hand, the selection and cultivation of highly productive forages (such as some C4 plants) showing optimal nutritional values is a strategy for ensuring fodder availability for livestock production economies, especially to satisfy the increasing demand due to global population growth, as stated by the Food and Agriculture Organization [6]. Moreover, the extended drought periods in temperate zones forecasted due to climate change are important aspects to take into account when designing an adequate strategy for fodder crop selection in Mediterranean climates. Therefore, C4 perennial and drought-tolerant plants (such as maralfalfa) can be good candidates against annual cereals to be introduced as fodder crops in Mediterranean areas, as they match both requisites of productivity and drought tolerance [7,8,9].

On the other hand, the quality of forages has to be assessed by measuring the chemical composition of those parameters related to the nutritional value. In this sense, the most important ones include the dry matter content (DM), ash, ether extract, EE (related to fat content), protein (CP), and fiber contents (both acid detergent fiber and ADF, and neutral detergent fiber or NDF), as well as the amino acids profile [10,11].

The nutritional parameters of forages (as happens with productivity) are influenced by several factors, including intrinsic features of the plant materials (genotypes, varieties, etc.), environmental conditions (climate, soils, etc.), and agronomic practices [9,12,13,14]. One of the most important agronomic practices is the determination of the optimal harvest time (HT), understood as the collection of biomass from forages at different periods or growth stages [14], as it strongly influences both the yields and nutritional quality of the fodder [15,16,17]. However, it is still not well known when to appropriately harvest maralfalfa to ensure optimum fodder production and nutritional value because, unlike other conventional forages in this climatic area, there are still few works of literature published on this species cropped under Mediterranean climates.

The productivity of maralfalfa is reported to be higher than 30 Tn/ha per year and up to 90 Tn/ha per year [5,18,19,20], which makes it quite suitable to be introduced as an industrial crop for various fields of application in several parts of the world. For instance, it was recently considered a good candidate for biofuel production by Nava-Berumen et al. [20]. The high dry matter yields and nutritive value of maralfalfa (high levels of proteins) also make this plant quite suitable to be used as a fodder crop [19]. In this regard, maralfalfa has been proposed as an alternative to other traditional forages in ruminating feeding such as alfalfa, barley, vetch, oat, or wheat [21], and the substitution of alfalfa (*Medicago sativa* L.) by maralfalfa has provided very good results in terms of forage quality (chemical composition and nutrient balance) and digestibility in Spain [22].

In tropical regions, the maralfalfa crop is harvested for around 180 days according to Calzada-Marin et al. [23], although the best plant materials for ruminant feeding are those obtained during the early cutting stages, where HTs between 40 and 75 days provided the best results [18]. However, the optimum HT can vary depending on the environmental conditions of a given region [15,16,17].

Taking into consideration the information stated above, the authors hypothesized that HT is a very important agronomic factor for ensuring the forage quality of maralfalfa, as it can have a strong impact on the nutritional quality of the plant. Therefore, the aim of this work was to investigate, for the first time, the effects of different HTs on the nutritional composition (DM, ash, EE, CP, NDF, and ADF) of maralfalfa (*C. purpureus*) cultured under Mediterranean climate, with a focus on the amino acids profile.

## 2. Materials and Methods

### 2.1. Experimental Crops and Plant Sampling

Maralfalfa was grown for 3 years in a one-hectare plantation established in April 2016 within the facilities of the company Biovic Consulting, S.L., located in Silla (Valencia, Spain) (Coordinates 39°37 N, −0°39 W). The experimental plots were established within a 3000 m^2^ area at the aforementioned plantation. Each experimental plot contained a 15 m^2^ row where plants were spaced by 0.75 m among them. Then, a uniform cutting was applied every year in April to homogenize the phenological stage of the plant before sampling in 2018. These plots were placed on the alluvial terraces with calcareous gleic fluvisols of alluvial and colluvial origin, typical of the marshes and borders that surround the rice crops in the Albufera Natural Park. The texture of these soils consisted of angular and heterometric detrital fragments embedded in silt-clayey matrices [24,25]. Plant material sampling was conducted at 8 HTs at intervals of 16, 28, 42, 58, 75, 87, 124, and 150 days after the uniformity-cutting practice (day 0) made on 25 April 2018. For each HT, plants were randomly collected from the plots in triplicate. The material was cut into small fragments and then mixed, and 1 kg of each was taken for further analysis. The experiment was extended up to 25 October 2018, and the overall duration of the experiments was 150 days. During this period, the plants were neither fertilized nor irrigated, and environmental conditions are shown in Table 1. The plant materials were immediately dried after sampling for 10 days at environmental conditions (20–22 °C) and then dehydrated in an oven at 50 °C for 72 h. Finally, the dried materials were stored in a low humidity atmosphere until the analytical determinations described below.

### 2.2. Ash

The ash was determined by placing 1–3 g of dry matter in a crucible and a muffle (Carbolite, Hope Valley, UK) at 550 °C for 6 h. Under these conditions, organic matter is eliminated by incineration. Ash measurement was performed using the gravimetric method according to Criscioni et al. [22] and following the recommendations of the Association of Official Analytical Chemists, AOAC [26].

### 2.3. Ether Extract (EE)

The determination of the ether extract (related to fat content) was carried out using the Soxhlet technique of solid-liquid extraction with diethyl ether and starting from 1–3 g of dry maralfalfa sample according to the methodology developed in [27]. A Soxhlet semiautomatic device (Foss 2050 Soxtec^TM^, Hillerød, Denmark) was used for these determinations.

### 2.4. Crude Protein (CP)

To estimate the total amount of nitrogen, the Kjeldahl quantitative method for total protein determination was employed using the Kjeldahl Foss Tecator 2006 (Foss Analytical, Hillerød, Denmark) according to the procedures described in [28]. In brief, 1–3 g of dried plant sample was prepared, and nitrogen was obtained using the correction factor (multiplication) of 5.83 [18]. The concentration of proteins in the extract supernatant was measured using the Bradford method [29] before and after precipitating by isoelectric point.

### 2.5. Fibers (Neutral, and Acid Detergent Fibers)

The structural polysaccharides (NDF and ADF) were determined following the procedures for fiber determination standardized by AOAC [26]. In brief, the procedure was performed as follows: firstly, the samples were treated with a neutral detergent solution, and starches, sugars, and pectins were solubilized by rinsing the samples with a heat-stable amylase. Secondly, hemicellulose was solubilized by using an acid detergent solvent, and the residue was treated with sulfuric acid (72% *w*/*v*) to dissolve cellulose. Finally, the measurement of NDF and ADF was made using the FT 122 Fibertec™ device (Foss Analytical, Hillerød, Denmark).

### 2.6. Plant Extraction and Crude Extract Purification

To obtain protein concentrates, the protocol developed by Uribarri et al. [30] for dwarf elephant grass was applied. This protocol involved homogenizing 15–30 g of dry plant material (previously ground to a size of 1 mm using a commercial grinder) with 100–200 mL of Ca(OH)_2_ to saturation using a homogenizer-disperser (Kinematica, Lucerne, Switzerland) in plastic cups. This mixture was then filtered using a Miracloth (Merck-Millipore, Darmstadt, Germany). The obtained extract was macerated in the cold for 24 h and further centrifuged for 30 min at 10,000 rpm in 50 mL centrifuge tubes to eliminate cell debris in the sediment. The obtained supernatant was used to purify the protein extracted, as described below.

### 2.7. Amino Acids Profile

The supernatant obtained in the previous section is precipitated by an isoelectric point according to [30] to obtain the protein extract. The procedure can be summarized as follows: the supernatant is brought to pH 4.5 with HCl and left in a water bath at 60 °C for 30 min. Then, the solution is left on ice for 15 min to force the precipitation of proteins present in the extract by isoelectric point (among other components of the supernatant). Two batches of 10–30 mg of this concentrate were weighed, and then subjected to two different procedures of hydrolyses. In the first one, acid hydrolysis was applied with 1.0 mL of 6N HCl in glass ampoules sealed with flame. Afterward, the sealed vials were left in an oven at 112 °C for 24 h. As tryptophan, methionine, and cysteine are degraded under these conditions [31], alkaline hydrolysis was also performed to have a complete amino acid profile. After hydrolysis, the glass vials were cooled and the contents were transferred to Eppendorf tubes. Then, the tubes were centrifuged at 10,000× *g* for 15 min, and the obtained supernatants were frozen at −80 °C and finally lyophilized. The final residue obtained was reconstituted in 1.5 mL of 0.1 N HCl to obtain a sample stock for chemical analyses. The AccQ-Tag pre-column amino acid derivatization method from Waters (Milford, CT, USA) was used before the identification of amino acids. The amino acid profile was studied using the High-Pressure Liquid Chromatography (HPLC) on a HITACHI ELITE LaChrom (Tokyo, Japan) device, equipped with a fluorescence detector. The AccQ-Tag amino acid analysis pre-column method from Waters (Milford, CT, USA) was used. In brief, 10–40 µL of the obtained sample stock was added to 960 µL (total volume of 1 mL) of ultrapure HPLC water. Aliquots of this solution were combined with the fluorescent reagent (6-aminoquinoline-N-hydroxysuccinimide carbamate) and other reagents in the kit (AccQ Tag Chemistry Package, Waters Co., Milford, CT, USA) following the manufacturer’s recommendations. The retention time for each amino acid was determined by using known calibration standards, prepared by mixing 40 µL of Waters Standard amino acid hydrolysate from a 2.5 mM stock prepared for each amino acid (Waters, Milford, CT, USA) in 960 µL of Ultra Water pure for HPLC, and processed as indicated for the samples. Amino acids were separated on a C18 reverse phase column (AccQ Tag, 3.9 × 150 mm, Waters Co, Milford, CT, USA) using a gradient of two eluents as mobile phase. Eluent A was prepared by adding 100 mL of buffer of concentrated acetate-phosphate (also present in the Waters kit) to 1 L of ultrapure water for HPLC. Eluent B consisted of 60% acetonitrile in ultrapure water for HPLC. The gradient used was the one recommended by the manufacturer with small modifications to optimize the separation of the amino acid peaks. The fluorescence detector was set at 250 nm of excitation and 395 nm of emission wavelengths. Finally, the data were processed with the EZChromeElite Software version 3.2.1 (Agilent, Santa Clara, CA, USA).

### 2.8. Determination of Tryptophan

A tryptophan standard was prepared at the concentration of 2.45 nM in 1 mL of 0.4 N NaOH. For the calibration line, seven dilutions were made from this stock solution (1/1, 1/2, 1/4, 1/8, 1/fifty). For the HPLC analyses, a mixture of 25 mM sodium acetate was prepared with ultrapure water, and 90% acetonitrile was used as an eluent under isocratic conditions and a flow of 1 mL/min. HPLC was prepared with the same C18 reverse phase column used for the separation of amino acids from acid hydrolysis, according to the Waters AccQ-Tag method described above. In this case, for tryptophan detection, a Photodiode Array absorbance detector was used and set at 280 nm. For quantification, the absorbance of the different tryptophan dilutions was measured. These were used to create the calibration. To calculate the amount of tryptophan present in the samples that were injected into the HPLC, calculations were made according to the equation:

y =7976.2x+90000006

where:

y = sample absorbance.

*x* = nanograms.

### 2.9. Experimental Design and Data Treatments

The data were collected from three replicates, and mean values were calculated. Before analyses, parameters expressed as percentages (ash, ether extract, crude protein content, neutral and acid detergent fibers) were transformed using the arcsine square root transformation to assure the homogeneity of variances, and therefore the normality of data. Data were statistically treated using one-way analyses of variance (ANOVA), and significant differences among means were stated using Tukey’s HSD (Honestly Significant Difference) test at the 95% level of confidence (α = 0.05). The results are presented as mean ± standard deviations. Data were also processed through a multivariate principal component analysis (PCA) to see whether relationships existed among the variables measured. All analyses were carried out using the software Infostat, version 2008 [32]. 

## 3. Results

The dry matter content showed an increasing trend with the culture period. The chemical composition of the tissues along the eight HTs showed different trends depending on the parameter considered (Table 2). The percentage of ash ranged from 6.42 ± 0.14 to 14.48 ± 0.21%. The highest percentages were recorded during the first four HTs and kept over 10% during this period (16, 28, 42, and 58 days) without significant differences among them. From the fifth HT (75 days) onward, the percentage of ash decreased by up to 6.80 ± 0.16%, showing significant differences when compared to the beginning of the experiment (in all cases, *p*-value < 0.0001). The CP ranged from 2.19 ± 0.11 to 21.57 ± 0.98%. Similar to what happened with ash, the CP showed a significantly decreasing trend over time (*p*-value < 0.0001). In this regard, the CP in the last HT (150 days) was almost seven times lower than the CP determined at the beginning of the experiment (16 days). On the contrary, both NDF and ADF increased almost linearly with the HT. NDF varied from 50.94 ± 0.42 to 78.94 ± 0.26%, whereas ADF changed from 30.12 ± 1.05 to 54.42 ± 0.41%. In contrast to all this, EE showed values lower than 0.10% in all cases and remained practically constant during the whole experimentation period, without significant differences among HTs.

The amino acid composition varied depending on the HT and the type of amino acid considered (Table 3). The most abundant non-essential amino acids were alanine, arginine, aspartate, and glutamic acid, which showed concentrations around 10% of the protein fraction in the eight HTs performed. Glycine and proline were present in moderate abundances—around 5% of the protein fraction—whereas the other non-essential amino acids were recorded at lower concentrations (histidine, serine, and tyrosine) or were not detected (cysteine). Among the essential amino acids, leucine, lysine, threonine, and valine showed the highest percentages of the protein fraction (around 6%), whereas phenylalanine and isoleucine showed slightly lower values (around 4–5%), and methionine and tryptophan were recorded at lower concentrations than 1% (Table 3). The concentrations of major-detected amino acids at different HTs showed an irregular trend, depending on the amino acid considered. For instance, alanine, arginine, proline, and leucine showed an increasing trend (with a maximum of 31.57 ± 9.21% at 124 days for alanine and 20.65 ± 4.83 at 75 days for arginine), whereas glutamic acid showed a decreasing trend. Other amino acids showed a more or less constant concentration over the experimentation period as it was recorded for aspartate, glycine, histidine, lysine, serine, threonine, and valine. In any case, the ratio of essential amino acids/total amino acids displayed more or less constant values of around 0.40 during the experimentation period, showing values ranging from 0.39 (16 days) up to 0.42 (150 days).

**Figure 1:**
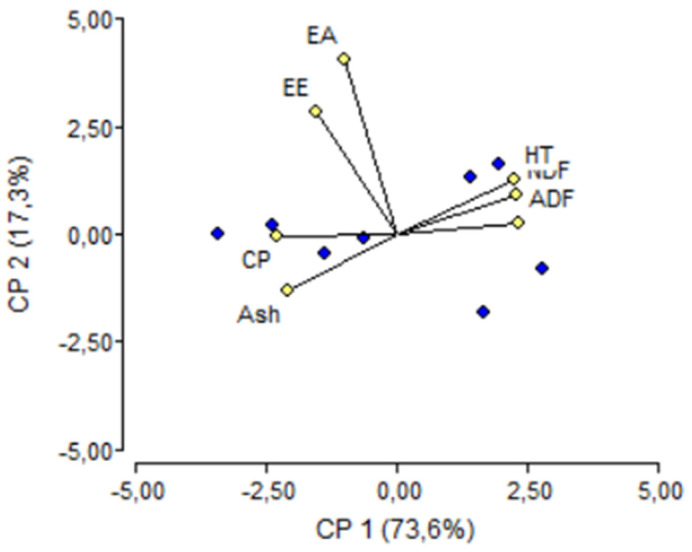
Biplot of Principal Component Analysis of the parameters studied: HT = harvest time; Ash = ash; EE = ether extract; CP = crude protein content; NDF = neutral detergent fiber; ADF = acid detergent fiber; EA = essential amino acids.

The results of the multivariate analyses (PCA) of the parameters measured are shown in Figure 1. Principal Component 1 (CP 1) contributed 73.6%, while Principal Component 2 (CP 2) contributed 17.3%, explaining 90.9% of the total variability. The latest HTs (5–8: from 75 to 150 days) are characterized by high levels of ADF and NDF, while the earliest HTs (1–4: from 16 to 58 days) are characterized by the highest levels of CP and ash. On the contrary, according to the biplot obtained, EE and EA remained uncorrelated to the HT.

## 4. Discussion

In the present work, the nutrient composition of maralfalfa was monitored at eight HTs over a 150-day cultivation experiment, comprising all stages of maximum vegetative growth of the cultivar. According to the results obtained, the nutritional composition displayed significant differences, showing that HT is a fundamental agronomic factor for fodder obtaining in livestock feeding.

The dry matter content, NDF, and ADF showed an increasing trend, whereas CP decreased over time, and EE, ash, and amino acids remained approximately constant. These controversial results open a dichotomy on what is the best harvesting period attending to the animal dry matter, nutrient intake, and digestibility as revealed in previous studies [14,33,34].

The percentage of dry matter content normally depends on the stage of growth; increases in this parameter are also described for other cereals, including the closely related hybrid *P. americanum* L. [8] and the elephant grass (determined as *P. purpureum* in [18,23]), where the authors found a positive correlation between the yield of stem biomass with the age of maralfalfa plants, similarly to what we obtained in the present work on dry matter content (minimum DM at 16 days = 13.00%, and maximum DM at 124 days = 25.20%). This increasing trend can be attributed to the leaf and stem development during vegetative growth, processes that imply higher biomass production as a result of the biosynthesis of structural and storage compounds before the flowering and fruiting stages, as described for other Mediterranean perennial fodder crops [23,35]. In the present work, the punctual increase in dry matter content observed at 124 days can be attributed to the rainfall period that occurred in June 2018 (see Table 1), similarly as observed at 150 days. The plant is therefore reactivated in response to natural irrigation, thus decreasing the dry matter content.

The CP ranged from 21.57% to 2.19% and values remained higher than 15% at the first two HTs (16 and 28 days). These values are in line with what is reported for other closely related cultivars (*P. americanum*) in different bioclimatic regions of the world [36], or even higher [8]. Also, this CP is higher than the CP reported for the other five varieties of oat (*Avena sativa* L.) cultivars [37], although these results are expectable as *A. sativa* is a C3 plant. In any case, the CP obtained in the present work can be considered high when compared to other cereals, and similar to other traditional forages in temperate areas such as alfalfa [22]. This fact makes maralfalfa interesting from a nutritional point of view, and suitable to be introduced as a fodder crop for livestock production. The CP decreased progressively over the culture period, and the most pronounced reduction occurred at the end of the experiment, at 124 days, which is not statistically different when compared to the last HT at 150 days (Table 2). This is also in line with the previous results published in other works performed on the genus *Pennisetum/Cenchrus* [8,18,19,30]. As can be observed, dry matter content is inversely related to the CP as described in wheat [14,38,39] and dicots such as in pea, *Pisum sativum* L. [40].

The amino acid composition (specifically, the profile in essential amino acids) contributes to the nutritive quality of the protein of forages for livestock feeding [41,42]. The profile of essential amino acids (see EA/TA relationship in Table 3) follows the trend described for the CP but in a more discrete manner, as it was observed after the third HT (42 days). However, the variability of the EA ratio can be considered to be constant during the experimentation period, as revealed in the biplot of the PCA performed (Figure 1). Similar values of EA/TA ratios were described for oat [37]. In the present work, the ratio of EA/TA remained more or less constant (Table 3). However, the quantity of a particular amino acid showed variations according to the HT. Among the essential amino acids, lysine and methionine are traditionally considered the most limiting ones in ruminants [43]. More recently, histidine has also been proposed as a limiting amino acid for ruminants in some circumstances [44]. The content of lysine measured in this work (around 6%) is similar to the content reported for other common cereal forages such as corn, sorghum, or wheat grains [43], or *Festuca arundinacea* Schreb., alfalfa, soybean meal, perennial ryegrass, and white clover, among other plant materials ([9], and references reviewed therein). It is twice as reported for the closely related *P. americanum* by Glew et al. [45], or five cultivars of oat [37], what makes maralfalfa quite interesting from the point of view of the EA profile, particularly lysine. The content of methionine is, however, sensibly lower than the values for all common forages reported in the [43], and also lower when compared to *P. americanum* [45], oat [37], *F. arundinacea*, alfalfa, soybean meal, perennial ryegrass, and white clover, among other plant materials ([9], and references reviewed therein). In any case, maximum values were obtained in the second HT (at 28 days), which reinforces our idea that the best HT is 28 days. In the case of histidine, values ranged from 1.48% to 3.64%. As happens with methionine, these histidine values are similar to those reported for other forages, such as *P. americanum* [45], oat [37], and *F. arundinacea* [9]. The real animal intake of these amino acids in forages cannot be ensured due to the interactions of proteins with the microbial population of the rumen, as well as the different enzymatic reactions that take place there [41,42]. This would limit the value of maralfalfa (and all forages in general) use due to its amino acid richness. In this regard, some strategies directed toward the rumen preservation of amino acids, such as microencapsulation [46], could be applied to preserve the forage quality of maralfalfa.

NDF and ADF show the composition of structural compounds of the plant cells (cellulose, hemicellulose, lignin, etc.). Low levels of these components are normally good indicators of the digestibility of the ingested forage, as high levels of NDF and ADF are digested slowly and remain in the animal rumen longer [33]. Therefore, both NDF and ADF are usually studied to establish the nutritional quality of the fodder as higher values of both fibers are related to lower digestibility of the ingested dry matter [14,33,34,47]. NDF ranged from ≈51% to ≈79% and ADF from ≈30% to ≈49% (Table 2), similar to what was reported in previous studies on *Pennisetum* and *C. purpureus* [8,36], maize [13], and wheat [14]. A practically linear increasing trend over the HTs was observed for both parameters in this study, where significant maximum levels were recorded after the fourth HT (58 days). This trend is related to the dry matter content as seen in the biplot performed (Figure 1), and it can also be attributed to the biosynthesis and accumulation of structural compounds during the vegetative growth of the plant, as we discussed above. In any case, the results of the NDF and ADF suggest that an early harvest of maralfalfa biomass is preferable to obtain a better performance of the livestock.

The results shown in the biplot of Figure 1 are in line with the ideas presented above and suggest that cutting age can be established at the second HT (28 days), as these HTs showed the optimal levels of nutritional parameters DM, ash, CP, EE, and EA in combination with moderate levels of non-digestible components such as NDF and ADF. Previous works performed on maralfalfa recommended HTs between 40 and 75 days ([18], and citations from that work]), which is longer than the results presented here. Therefore, a harvesting strategy based on an early HT is optimal to obtain the best equilibrium in terms of nutritional quality of the forage.

Finally, this work establishes the basis for the optimal HT of maralfalfa under a Mediterranean climate to achieve the best forage quality in a non-fertilized and non-irrigated experimental crop. The results provided here showed that maralfalfa is a promising fodder crop due to its richness in proteins and amino acids, but further research is needed to optimize the use of this plant for livestock production in Mediterranean countries. In this regard, the optimization of its nutritional properties, taking into consideration other agronomic practices (nitrogen fertilization, irrigation, etc.), must be the next step. As nitrogen fertilization is often related to a higher dry matter content—and higher levels of protein, amino acids, and nucleic acids, among other primary and specialized metabolites with a crucial role in plant development [8,22,48,49,50,51,52]—further works performed on maralfalfa should be directed to study the effects of various fertilization regimes on nutritional parameters within the optimal HTs determined in the present work. Finally, the increasing use of wild medicinal plants as health promoter agents in sustainable livestock production has been recently highlighted [53,54,55]. Plant extracts and herbal mixtures containing specialized metabolites like phenolic acids, flavonoids, and terpenes (among others), are widely used in feed additives for rumen manipulation (phytogenics), and they display an interesting potential in animal nutrition for livestock production and reproduction [55]. Given the large number of references on the traditional use of wild medicinal plants as forages [56,57,58,59], and the role of the plant tissue culture techniques in producing chemically stable plants at the industrial scale [60,61,62], it will certainly be interesting to investigate the nutritional value of maralfalfa mixtures containing these medicinal plants to take the most advantage of this promising forage in sustainable livestock feeding in Mediterranean climates.

## Figures and Tables

**Table 1:** Monthly mean temperature (°C) and total rainfall (mm) recorded during the years 2017 and 2018 according to the official climate information data available for La Pilotera de Silla (www.avamet.org; accessed on 15 September 2023).

	Temperature 2017 (°C)	Rainfall 2017 (mm)	Temperature 2018 (°C)	Rainfall 2018 (mm)
January	9.3	92.1	14.0	29.1
February	11.8	14.7	12.5	58.8
March	18.3	48.1	16.8	13.6
April	17.6	34.6	18.1	18.3
May	20.8	4.2	20.3	21.4
June	25.2	42.8	24.4	90.9
July	26.0	0.6	27.2	2.4
August	27.5	18.1	27.4	2.2
September	26.2	13.4	24.2	186.3
October	21.4	21.7	18.9	227.9
November	14.6	7.4	15.0	154.7
December	9.3	3.8	13.1	12.0

**Table 2:** Chemical composition (Ash, EE: ether extract, CP: crude protein content, NDF: neutral detergent fiber, ADF: acid detergent fiber) of maralfalfa (*Cenchrus purpureus* (Schumach.) Morrone) along 8 harvest times (HTs) during the maximum vegetative growth of the crop (April–October, 2018).

HTs (Days)	DM (%)	Ash (%)	EE (%)	CP (%)	NDF (%)	ADF (%)
16	13.00	13.13 ± 0.32 ab	0.019 ± 0.003 a	21.57 ± 0.98 a	50.94 ± 0.42 g	30.12 ± 1.05 f
28	16.40	13.07 ± 0.17 ab	0.016 ± 0.002 a	15.53 ± 0.71 b	57.13 ± 1.11 f	34.76 ± 0.38 e
42	15.40	14.48 ± 0.21 a	0.014 ± 0.012 a	8.84 ± 0.12 c	63.12 ± 0.62 e	37.52 ± 1.03 d
58	19.40	13.38 ± 0.12 ab	0.018 ± 0.01 b	6.49 ± 1.33 d	67.32 ± 0.88 d	41.86 ± 0.69 c
75	16.60	09.24 ± 0.39 bc	0.001 ± 0.0002 a	3.91 ± 0.12 e	71.35 ± 0.59 c	49.16 ± 0.48 b
87	13.80	7.08 ± 2.90 c	0.017 ± 0.010 a	3.88 ± 0.14 e	75.72 ± 0.59 b	52.87 ± 0.16 a
124	25.20	6.42 ± 0.14 c	0.008 ± 0.001 ab	2.19 ± 0.11 ef	78.24 ± 0.64 a	54.42 ± 0.41 a
150	16.50	6.80 ± 0.16 c	0.009 ± 0.002 ab	3.08 ± 0.01 f	78.94 ± 0.26 a	49.06 ± 0.80 b

Data are presented as means ± standard deviations from 3 replicates. Different letters within columns indicate significant differences according to Tukey’s HSD test at the 5% level of significance.

**Table 3:** Amino acid composition (% of the protein fraction) of maralfalfa (*Cenchrus purpureus* (Schumach.) Morrone) along harvest times (HTs) during the maximum vegetative growth of the crop (April–October 2018).

Amino Acid	HTs (Days)							
	16	28	42	58	75	87	124	150
Alanine	7.29 ± 0.70	8.84 ± 1.47	7.50 ± 0.85	16.77 ± 2.12	17.33 ± 2.92	13.24 ± 4.09	31.57 ± 9.21	8.60 ± 2.49
Arginine	10.12 ± 0.31	9.72 ± 2.47	11.18 ± 1.76	7.59 ± 0.49	20.65 ± 4.83	11.37 ± 1.24	6.47 ± 1.31	8.92 ± 1.18
Aspartate	11.21 ± 0.09	10.67 ± 0.24	10.46 ± 0.26	11.95 ± 0.57	8.69 ± 1.82	8.94 ± 1.18	8.63 ± 1.82	11.53 ± 0.37
Cysteine	0.00 ± 0.00	0.00 ± 0.00	0.00 ± 0.00	0.00 ± 0.00	0.00 ± 0.00	0.00 ± 0.00	0.00 ± 0.00	0.00 ± 0.00
Glutamic acid	12.13 ± 0.08	12.23 ± 0.24	12.49 ± 0.20	11.00 ± 0.45	4.34 ± 0.30	5.26 ± 0.60	5.29 ± 0.90	7.34 ± 0.24
Glycine	4.51 ± 0.07	4.80 ± 0.19	3.78 ± 0.04	4.14 ± 0.08	4.02 ± 1.43	4.68 ± 0.67	3.96 ± 0.64	4.58 ± 0.33
Histidine	2.43 ± 0.09	1.71 ± 0.15	1.68 ± 0.22	1.48 ± 0.11	2.18 ± 0.30	3.64 ± 0.13	2.20 ± 0.44	3.37 ± 0.39
Isoleucine *	4.82 ± 0.12	4.95 ± 0.10	5.13 ± 0.08	4.80 ± 0.13	4.07 ± 0.37	4.96 ± 0.60	4.22 ± 0.87	5.21 ± 0.18
Leucine *	7.82 ± 0.15	8.17 ± 0.14	8.53 ± 0.12	7.80 ± 0.23	7.16 ± 1.58	7.83 ± 0.97	6.45 ± 1.35	8.98 ± 0.05
Lysine *	5.90 ± 0.01	6.00 ± 0.09	6.23 ± 0.24	5.39 ± 0.15	3.29 ± 1.38	6.14 ± 0.62	3.39 ± 1.49	5.65 ± 0.46
Methionine *	0.61 ± 0.06	0.02 ± 0.04	0.57 ± 0.42	0.21 ± 0.03	0.00 ± 0.00	0.00 ± 0.00	0.00 ± 0.00	0.00 ± 0.00
Phenylalanine *	4.93 ± 0.17	5.18 ± 0.07	5.18 ± 0.07	4.95 ± 0.15	4.39 ± 0.64	5.08 ± 0.37	4.47 ± 0.96	5.79 ± 0.14
Proline	5.93 ± 0.15	5.98 ± 0.20	5.45 ± 0.25	6.17 ± 0.37	5.15 ± 0.40	5.86 ± 1.03	6.11 ± 1.31	7.45 ± 2.58
Serine	4.62 ± 0.59	3.77 ± 0.01	3.73 ± 0.18	3.35 ± 0.21	3.27 ± 0.36	3.95 ± 0.46	3.53 ± 0.73	4.66 ± 0.12
Threonine *	7.89 ± 0.07	7.81 ± 0.04	7.73 ± 0.06	6.08 ± 0.27	7.00 ± 0.17	8.03 ± 1.17	5.45 ± 1.23	7.47 ± 1.20
Tryptophan *	0.52 ± 0.09	0.49 ± 0.09	0.38 ± 0.07	0.00 ± 0.00	0.00 ± 0.00	0.03 ± 0.02	0.07 ± 0.03	0.09 ± 0.04
Tyrosine	2.37 ± 0.10	2.29 ± 0.97	2.75 ± 0.91	1.43 ± 0.15	2.13 ± 0.31	3.78 ± 0.77	2.04 ± 0.78	2.03 ± 0.22
Valine *	6.91 ± 0.17	7.37 ± 0.23	7.24 ± 0.24	6.86 ± 0.27	6.43 ± 0.76	7.21 ± 0.42	6.16 ± 1.48	8.39 ± 0.27
essential aa/ total aa	0.39	0.4	0.36	0.36	0.32	0.39	0.3	0.42

Data are presented as means ± standard deviations. * Indicates those essential amino acids.

## Data Availability

The data that support the findings of this study are available from the corresponding author, J.C.G., upon reasonable request.
